# SARS-CoV-2–Specific Vaccine Candidates; the Contribution of Structural Vaccinology

**DOI:** 10.3390/vaccines10020236

**Published:** 2022-02-03

**Authors:** Su Min Pack, Peter J. Peters

**Affiliations:** The Maastricht Multimodal Molecular Imaging Institute (M4i), Faculty of Health, Medicine and Life Sciences (FHML), Maastricht University, 6229 ER Maastricht, The Netherlands; qortnals97@gmail.com

**Keywords:** structural vaccinology, COVID-19, vaccine, SARS-CoV-2, modern vaccine, S protein, S1 subunit, S2 subunit, pan-coronavirus vaccine, broad-spectrum vaccine, nucleic acid vaccine, recombinant protein vaccine, mRNA vaccine, vectored vaccine, receptor binding domain

## Abstract

SARS-CoV-2 vaccine production has taken us by storm. We aim to fill in the history of concepts and the work of pioneers and provide a framework of strategies employing structural vaccinology. Cryo-electron microscopy became crucial in providing three-dimensional (3D) structures and creating candidates eliciting T and B cell-mediated immunity. It also determined structural changes in the emerging mutants in order to design new constructs that can be easily, quickly and safely added to the vaccines. The full-length spike (S) protein, the S1 subunit and its receptor binding domain (RBD) of the virus are the best candidates. The vaccine development to cease this COVID-19 pandemic sets a milestone for the pan-coronavirus vaccine’s designing and manufacturing. By employing structural vaccinology, we propose that the mRNA and the protein sequences of the currently approved vaccines should be modified rapidly to keep up with the more infectious new variants.

## 1. Introduction

Emerging 17 years ago [1], severe acute respiratory syndrome coronavirus 1 (SARS-CoV-1) is a coronavirus belonging to a group of enveloped viruses with a positive-sense, single-stranded RNA (RNA (+)) genome and a nucleocapsid. Their name is derived from the characteristic club-shaped spikes that project from their surface; these spikes resemble a solar corona when the virus particle is visualised with an electron microscope [2]. In December 2019, SARS-CoV-2 emerged in Wuhan, China [3]. This novel virus has a higher reproductive number of infections compared to previous coronaviruses [4], which eventually resulted in the outbreak of the COVID-19 pandemic. The virus causes symptoms such as severe pneumonia and multi-organ failure, which may lead to death [5]. In addition, the virus also has had an enormous impact on the economy, politics and society. Worldwide, the race to design the most variant-updated, immunogenic and massively producible COVID-19 vaccine is still ongoing.

The remarkable quality and speed of COVID-19 vaccine development were possible thanks to the aggregation of three decades of scientific progress in the fields of reverse vaccinology, structural vaccinology, synthetic biology and vaccine adjuvants [6]. Upon the release of the SARS-CoV-2 genetic sequence data by a group of scientists from Fudan University, China, in January 2020, many laboratories were already equipped with the technology to create the vaccines from the synthetic genes [7]. Facilitated by new structural insights of the viral proteins gained through structural vaccinology tools, the world’s first commercialised mRNA vaccine was released by Pfizer-BioNTech in December 2020 [8]. Other COVID-19 vaccines employing the modern vaccine platforms quickly followed, and many more are under clinical trials [9].

Here, we review the development of the World Health Organisation (WHO)-approved modern SARS-CoV-2 vaccines with the emphasis on the importance of structural vaccinology in enabling the vaccine design with unprecedented speed. We address the potential of a complete spike (S) protein and its sub-domains as suitable SARS-CoV-2–specific and/or pan-coronavirus vaccine candidates. The importance of structural vaccinology, the employment of modern vaccine platforms and insights on designing potent coronavirus vaccines will be highlighted.

## 2. SARS-CoV-2 Infection

**Figure 1:**
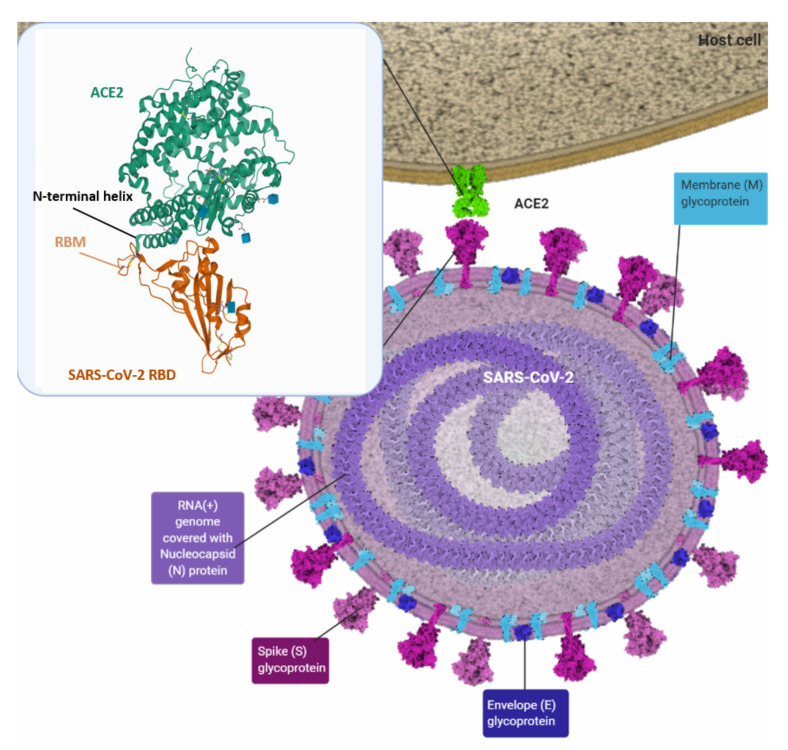
Structure of SARS-CoV-2 virus and receptor binding domain (RBD)–angiotensin-converting enzyme 2 (ACE2) interaction using cryo-EM. Host ACE2 and viral RBD interaction observed using cryo-EM. ACE2 depicted in green, SARS-CoV-2 RBD core in brown and receptor binding motif (RBM) within RBD in orange. Created with CellPAINT 2.0 and BioRender. Image from the RCSB PDB (rcsb.org) of PDB ID 6M0J (Wang, X., Lan, J., Ge, J., Yu, J., Shan, S.) (2020) Crystal structure of SARS-CoV-2 spike receptor-binding domain bound with ACE2. Available from: https://doi.org/10.2210/pdb6m0j/pdb (accessed on 26 January 2022). Created with CellPAINT 2.0 and BioRender.

During infection, the SARS-CoV-2 virus is endocytosed into the host cell, initiated by the interaction between viral trimeric S protein and the host cell-surface receptor angiotensin-converting enzyme 2 (ACE2) (Figure 1) [10]. In addition to the lung and respiratory tract, ACE2 receptors are also highly expressed in other organs such as the small intestine, testis, kidney, heart muscle, and colon, explaining why SARS-CoV-2–infected patients encounter gastrointestinal problems and kidney dysfunction [11]. SARS-CoV-2 is known to have a 10- to 20-fold higher binding affinity to the ACE2 receptor than SARS-CoV-1, partly justifying its enhanced pathogenicity and infectivity compared to SARS-CoV-1 [12]. The densely glycosylated S protein consists of two subunits, S1 and S2, created by a furin-like protease [4]. The receptor-binding domain (RBD) within the S1 subunit recognises and binds to the ACE2 receptor, resulting in the S1 subunit shedding and consequentially stabilising the post-fusion conformation of the S2 subunit [12]. The RBD oscillates between ‘up’ and ‘down’ conformations, transiently exposing itself only in the ‘up’ conformation to interact with the ACE2 receptor [12]. The S2 subunit is thereafter cleaved by host proteases, for instance, transmembrane protease serine 2 (TMPRSS2) [13], to undergo extensive irreversible conformational changes, which are essential for fusion of the viral membrane and host plasma membrane to facilitate the release of viral RNA into the host cytoplasm [10].

## 3. Structural Vaccinology

Structural vaccinology is a designing strategy that often comes hand-in-hand with reverse vaccinology, which screens for potential target antigens from the viral genome that can be recognised by antibodies (Abs) and receptors on immune cells [14]. These antigenic target proteins, such as the S protein of the SARS-CoV-2, normally engage in critical interactions with its host receptor(s) for viral invasion [10]. Abs generated by the host B cells interacting with various epitopes on these target proteins perturb the pathogen–host cell interaction, leading to infection failure and/or clearance of the pathogen [15]. After the selection of potential antigens, structural vaccinology tools such as X-ray crystallography, nuclear magnetic resonance (NMR) spectroscopy and cryo-electron microscopy (cryo-EM) are employed to determine the detailed, three-dimensional (3D) atomic structure of the antigens and antigen–Ab complexes. For COVID-19 vaccine development, cryo-EM was used to reveal the dynamic state of S protein domains and analyse the S protein subunit conformation transformation upon docking with the host ACE2 receptor during viral entry [10,12].

In order to define the tertiary and quaternary structures of a viral protein in general, the atomic coordinates of multiple protein subunits including its protective sugar chains of the virus spike, the host receptor–epitope interaction, antigen structure and antigen–Ab complexes provided by structural biology techniques must be examined [14]. This information, together with knowledge of the pathogen genome, its amino acid mutations [16], 3D protein conformations and stability and immunological effects [14], is vital for efficient identification, selection and modification of the antigens and/or epitopes [14,17] in their most favourable positions, orientations and stability [18].

**Figure 2:**
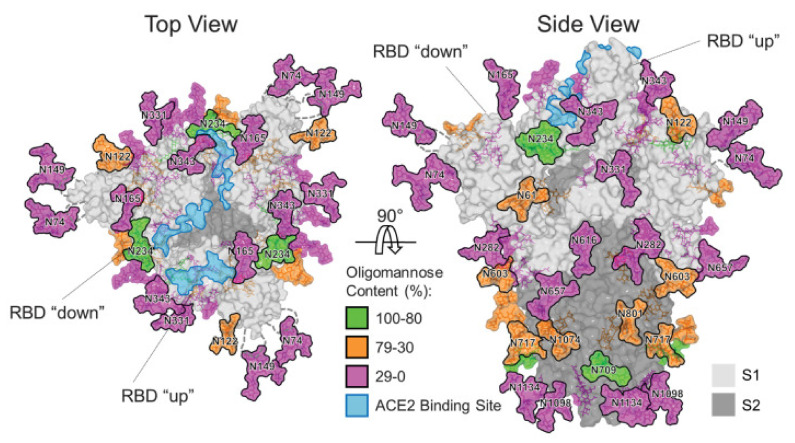
Structural-biology-based glycosylation map of the trimeric S protein of SARS-CoV-2. The green, orange and pink regions represent different amounts of high-mannose-type glycans shielding regions of S protein. The blue regions are the host ACE2 binding site essential for virus entry into the host cell. The figure also illustrates the ‘up’ conformation and ‘down’ conformation of two of the three receptor binding sites (RBD), which is only accessible for ACE2 or antibody binding when it is in ‘up’ conformation. Reprinted from Watanabe et al. [20]. The permission to reprint was granted by PMC.

With the structural and immunological information, many investigations, including those conducted by Wrapp et al. and Pinto et al., proposed the S protein as a suitable vaccine candidate to evoke appropriate immunity [12,19]. The structure of the SARS-CoV-2 glycosylated S protein was also solved by Watanabe et al. using cryo-EM (Figure 2) [20]. High-mannose-type glycans shield the viral epitopes on the S protein from the host immune cells, resulting in immune evasion of the virus; the degree of glycan shielding is correlated to viral immune evasion. Based on cryo-EM data, Walls et al. speculated that the furin-cleaved site in the S protein boosted the tropism and transmissibility of the SARS-CoV-2 virus, enhancing its pathogenicity in comparison to previous pathogenic human coronaviruses [10]. Only the exposed unglycosylated regions can be recognised by the host immune cells to stimulate anti-viral T cell immunity and/or B cells to produce antigen-specific neutralising (n) Abs. These regions are superb vaccine targets as they can be engaged by the nAbs to disrupt the S protein–ACE2 receptor association, hindering viral entry and stimulating Ab-mediated viral clearance [15].

Structural vaccinology also enables modifications to be made to improve vaccine candidates. For example, the amino acid sequences of antigens are often synthetically mutated to improve their stability to elicit appropriate antiviral immunity, mask unwanted non-neutralising Ab epitopes, optimise vaccine thermostability and synthesise multi-epitope vaccines. For COVID-19 vaccines, the determination of the atomic structure of S protein subunits using structural vaccinology tools enabled researchers to generate pre-fusion stabilised SARS-CoV-2 protein, which is more stable and expressed at a higher level in comparison to the wild-type soluble S protein [21]. Juraszek et al. modified heptad repeat (HR) region 1 of the S2 subunit and subunit domain 1 at the interface with S2 and achieved 6.4 times enhanced expression of the stabilised S protein trimer [22]. They employed high-resolution cryo-EM to identify stabilising mutations in S1 and S2 subdomains, therefore designing a highly stable, correctly folded S trimer that is predominantly in closed pre-fusion conformation. These mutated S proteins remained stable in the absence of a heterologous trimerisation domain, which is required in soluble S proteins generally. Additionally, a group of scientists from Pfizer and BioNTech stabilised the pre-fusion state of the S protein by introducing mutations in S residues 986 and 987 to prolines, giving the name P2 S protein [23]. Its structure was confirmed by cryo-EM. They demonstrated that the S protein captured in the pre-fusion conformation elicited authentic SARS-CoV-2 RBD nAb in rhesus macaques, with titres 10.2- to 18.0-fold higher than when SARS-CoV-2 convalescent human serum was injected. This mutated trimeric S protein exhibits permanent one ‘up’ and two ‘down’ state RBDs, resulting in the continuous exposure of neutralising epitopes for immune cell recognition compared to the wild-type S protein, which exposes the Ab-targetable RBD epitopes transiently. With further development, this mRNA vaccine encoding the P2 S pre-fusion conformation became the world’s first US Food and Drug Administration (FDA)-approved mRNA vaccine [24]. In another approach to stabilise the pre-fusion S protein and improve protease resistance, a supplementary 682-QQAQ-685 mutation at the S1–S2 cleavage site was made [25].

Employing cryo-EM to study antigen–antibody interaction also facilitated the identification of novel antigens as potential vaccine candidates. By studying the RBD-H104 immunoglobulin (Ig) G interaction and affinity, Lv et al. discovered several epitopes that are identical between SARS-CoV-1 and SARS-CoV-2 viruses [26]. They suggested the possibility of developing a pan-coronavirus vaccine. Furthermore, Barnes et al. proposed different classes of nAb, which do not overlap with each other, binding specifically to various RBD epitopes [27]. If these epitopes are targeted by vaccine or Ab-based therapeutic intervention, cross-neutralisation of coronavirus strains could be expected.

Structural vaccinology and bioinformatic analysis are excellent tools for vaccine design [14]: They provide critical structural details of antigens to further modify and manipulate the vaccine candidates to enhance their efficacy and safety [17]. The re-engineered antigen-expressing genomic material or remodelled antigens in a well-folded 3D protein complex are then incorporated into a vaccine platform (be it its mRNA, vector enclosed or recombinant protein) to be tested for safety and efficacy in animal models [14]. The vaccine candidates then undergo preclinical and clinical trials to evaluate their immunogenicity, antigenicity, stability and safety. Based on the trial results, the vaccine candidates can be further optimised using structural vaccinology.

## 4. SARS-CoV-2 Spike Protein

Many researchers have pinpointed the SARS-CoV-2 whole S protein or S protein subunits as ideal vaccine targets based on structural studies [4,28]. The S protein is an attractive vaccine target as it is the key determinant to the tropism and pathogenicity of the virus [10,29] and is capable of inducing strong anti-viral immunity to produce high nAb titre [16,30], blocking viral invasion into the host cells [4,28].

The S homotrimer protein consists of a membrane-distal S1 subunit and a membrane-proximal S2 subunit and exists on the viral envelope [31]. The S1 subunit is vital for the host ACE2 receptor recognition via its RBD, and the S2 subunit is responsible for membrane fusion, crucial for virus entry into the host cell. Upon recognition of the host ACE2 receptor, the S protein undergoes a conformation change from pre-fusion to a more stable post-fusion state, shedding the S1 subunit while refolding the S2 subunit to facilitate membrane fusion [12,21]. Similarly, the synthetic recombinant S protein tends to metastasise and transform into its post-fusion conformation to dissociate the S1 subunit. However, this is not favourable as the S1 subunit consists of numerous immunodominant epitopes that can be targeted and neutralised by Abs, serving as a potent vaccine target [32]. Indeed, the P2 S proteins of MERS-CoV, SARS-CoV-1 and HKU1 in their pre-fusion conformations were described as antigenically optimal [33] and elicited much higher nAb titres than the wild-type S proteins in a mouse model. Therefore, strategies to stabilise the S protein in its pre-fusion state were investigated in SARS-CoV-2 vaccine development. Pfizer-BioNTech’s BNT162b2, Moderna’s mRNA-1273, Janssen Pharmaceutical’s Ad26.COV2.S and Novavax’s NVX-CoV2372 vaccines are based on the full-length P2 S glycoprotein (Table 1) [34].

## 5. Modern Vaccine Platforms

Modern vaccine platforms include vectored, nucleic acid and recombinant protein vaccines. COVID-19 vaccines targeting the P2 S protein include all three platforms. A vectored vaccine refers to any genomic material in a viral vector [35] that can be replication-incompetent, replication-competent or inactivated. Although there are distinct pros and cons of each of these, the vectored vaccines developed in the past year, Oxford-AstraZeneca’s ChAdOx1nCoV-19 and Janssen’s Ad26.COV2.S, both employ genetically altered replication-incompetent vectors (Table 1) [35]. These vaccines are designed as intramuscular vaccines, endocytosed by cells, translocated to the cytosol to transcribe and express SARS-CoV-2 S protein. A single dose of Ad26.COV2.S, a vaccine with a recombinant replication-incompetent human adenovirus vector, protected against both symptomatic and asymptomatic SARS-CoV-2 infection. It demonstrated 85.4% and 93.1% efficacy against severe disease and hospitalisation, respectively [36]. Both arms of adaptive immunity are stimulated when the cells present the S protein to the respective immune cells. One of the common concerns regarding vectored vaccines is the induction of pre-existing immune response against the viral vector itself, instead of the antigen encoded in the genetic material, resulting in the neutralisation of the vector [37]. This response may reduce vaccine efficiency. However, this problem is addressed by using vectors that are rare in humans or only infect animals. For instance, the Oxford University research team involved in the ChAdOx1nCoV-19 vaccine development reported that the levels of pre-existing ChAdOx1 viral vector Ab in human samples were significantly lower than the published data for other chimpanzee adenovirus vectors [37].

The COVID-19 vaccines currently given the most attention are nucleic acid-based vaccines. Nucleic acid vaccines can be produced completely in vitro, enabling rapid synthesis to meet the large quantity urgently needed for COVID-19 [38]. Of the two types of nucleic acid vaccines, mRNA vaccines are technically favoured over DNA vaccines [39]. DNA vaccines must be transported into the nucleus for antigen transcription before translation in the cytosol, whereas mRNA vaccines only need to cross the plasma membrane for antigen synthesis [40]. Additionally, DNA vaccines can theoretically be integrated into the host genome, giving rise to the risk of insertional mutagenesis [41]. However, mRNA vaccines are designed for cytosolic delivery and lack specific sequence motifs for nuclear targeting, hence there is no risk of genome integration [39]. They are also expressed in a very low concentration in the cytosol and have a short half-life, resulting in a more controlled antigen expression [42,43]. The BNT162b2 vaccine developed by Pfizer-BioNTech and the mRNA-1273 vaccine designed by Moderna, both mRNA vaccines, were granted Emergency Use Authorization by the FDA within one year after the outbreak of the pandemic [44]. Eventually, the BNT162b2 vaccine was approved as the first COVID-19 vaccine and the first human mRNA vaccine in August 2021 by the FDA within 2 years after the outbreak of the pandemic [24]. However, long-term storage stability may be a problem for this vaccine platform. Pfizer’s BNT162b2 vaccine requires storage at −70 °C, while Moderna’s mRNA-1273 can be stored for 6 months at −20 °C and in the refrigerator at 2–8 °C for 30 days [45,46]. This cold-chain requirement to maintain vaccine efficacy may hinder the distribution and eventually add further burden to vaccine waste [47].

The third modern vaccine platform is recombinant protein vaccines. Against the SARS-CoV-2 virus, the recombinant S protein or the RBD are reversely and structurally engineered to elicit appropriate host immune responses in order to neutralise and prevent the virus from docking on the host ACE2 receptor for viral replication [48]. The full-length recombinant SARS-CoV-2 S glycoprotein nanoparticle vaccine, NVX-CoV2373, designed by Novavax, is one of the earliest vaccine candidates enrolled in clinical trials (Table 1) [9]. Novavax’s recombinant protein vaccine formulated with the adjuvant Matrix M is expressed in insect cells with a baculovirus system. This vaccine successfully demonstrated Th1-biased immune response and induction of high nAb titres after two injections of two different doses, achieving more than 90% protective efficacy against the original coronavirus strain from Wuhan, China [49]. In Dec 2021, Novavax’s vaccine became the first COVID-19 recombinant protein vaccine conditionally approved by the European Medicines Agency (EMA) [50]. Although recombinant protein vaccines require significantly more time for development and production, they provide some distinct advantages over viral vector and nucleic acid vaccines. First, recombinant protein vaccines trigger a safe and robust immune response against pathogens, as proven by the hepatitis B vaccine [51]. Furthermore, unlike nucleic acid vaccines that require advanced production facilities and cold-chain transport systems, facilities and resources for the production, the transport and storage requirements of recombinant protein vaccines are readily available and less demanding [52]. These characteristics facilitate distributing vaccines to low- and middle-income countries. Finally, recombinant protein vaccines do not face pre-existing immune responses against the viral vector, as is problematic in viral vector vaccines.

Modern vaccine platforms have disadvantages and advantages over traditional inactivated virus or live-attenuated virus vaccines [51]. One disadvantage may be the poorer immunogenicity of simpler vaccine antigens, which can be ameliorated by the incorporation of adjuvants [16]. The BNT162b2 mRNA vaccine for SARS-CoV-2 is adjuvanted with lipid- or polymer-based nanoparticles to stabilise and prevent degradation of mRNA and enhance its uptake by the myocytes and/or infiltrating dendritic cells at the site of injection [53]. Furthermore, innate immune recognition of mRNA has been proven to enhance immunogenicity to a degree that is comparable to adjuvant effects. The BNT162b2 mRNA vaccine reported 95% protective efficacy [54]. As one advantage, vectored and nucleic acid vaccines can mimic an actual infection by inducing both CD8+ and CD4+ T cell-mediated immunity, allowing synergetic T and B cell-mediated immunity to fight viral infection. Together with the well-studied B cell-mediated immunological memory, T cell memory may be critical for prophylactic measures against infectious diseases [55]. Designing modern vaccines capable of eliciting both arms of immunity by employing reverse engineering and structural vaccinology tools may pave way for more virus-specific protection with a lower risk of adverse effects.

Despite the high protective efficacy exhibited by existing SARS-CoV-2 vaccines involving the P2 S protein, concerns regarding the possibility of an S immunogen-evoked antibody-dependent enhancement (ADE) and other adverse effects were raised [28]. ADE refers to a phenomenon in which virus-specific, non-neutralising Abs mediate the virus entry and/or replication into host cells via interactions with host receptors, instead of blocking or clearing the infection [56]. Although there are currently no verified reports of COVID-19 vaccine-induced ADE cases, earlier studies confirmed that ADE of SARS-CoV-1 and MERS-CoV can occur due to ineffective Abs [57,58]. Additionally, Maemura et al. discovered through in vitro experiments that the ADE of SARS-CoV-2 infection may be facilitated by the IgG receptors FcγRIIA and FcγRIIIA [59]. These studies alert us of the possible risk of SARS-CoV-2 infection and/or vaccine-induced ADE. In the US, 11.1 anaphylaxis cases per million doses of the BNT162b2 mRNA vaccine were reported [60]. Even though the risk of life-threatening allergic reactions induced by the vaccines are unlikely, securing necessary resources to manage the side effects is vital to ensure both the safety and efficacy of the vaccines.

## 6. Alternative Candidates for SARS-CoV-2 Vaccines

While all approved COVID-19 vaccines target the full-length S protein (Table 1), other targets have been identified. Two such candidates are in the S1 subunit: the receptor binding domain (RBD) and the N-terminal domain (NTD). Within the SARS-CoV-2 S glycoprotein, the RBD is known to be an important vaccine target to elicit humoral immunity as strong as [28,61] or even stronger than the full-length S protein [30]. The RBD consists of epitopes that induce high levels of S-specific nAb production and Th1-biased responses. Additionally, the RBD is a highly conserved structure among various coronaviruses, and RBD-based antigens have been described as the primary candidate for the SARS-CoV-1 and MERS-CoV vaccines [34,62]. The nAb against the SARS-CoV-1 RBD successfully hindered S1–ACE2 interaction, decreased the viral load significantly in all infected mice [63] and protected up to 12 months after immunisation [28]. Moreover, the RBD-based vaccine is believed to be less potent to arouse immunopathogeneses. While the surface of the S protein is extensively shielded with glycans (Figure 2) that prevent Ab recognition, the RBD is largely accessible to its specific nAb [64]. This accessibility explains the immunodominance of RBD epitopes and suggests that the shorter protein subunit vaccines may confer to better safety profile than vaccines of the full-length S protein vaccine [37] which contains epitopes that may stimulate ADE, consequentially aggravating the infection [65].

For SARS-CoV-2, Jiang et al. analysed the RBD-specific Ab response from a panel of sera from animals immunised with RBD-based antigens and identified four linear B cell epitope peptides: 350VYAWN345, 407VRQIAP412, S450-469 and 473YQAGSTP479 [66]. Three immunodominant peptides, 350VYAWN345, 407VRQIAP412 and 473YQAGSTP479, were revealed to induce a potent Ab response to the SARS-CoV-2 S protein in the mouse model. The researchers identified that the 350VYAWN345 epitope sequence was highly conserved among different SARS-CoV-2 strains. Additionally, 407VRQIAP412 shared cryptic epitopes between SARS-CoV-1 and SARS-CoV-2. Anhui Zhifei Longcom Biopharmaceutical Co., Ltd., is currently designing a SARS-CoV-2 vaccine antigen based on an RBD dimer produced in mammalian cells [67]. The scientists demonstrated that this dimerisation increases the stability of the vaccine antigen for SARS-CoV-2, SARS-CoV-1 and MERS-CoV constructs. Both studies argue for designing an RBD-specific vaccine.

In addition to the B cell response against SARS-CoV-2, T cell responses can theoretically be anticipated by the RBD-based vaccine. Of the 20 T cell epitopes identified in the SARS-CoV-2 S protein, 9 epitopes were found in the RBD [68]. Other than the direct cytotoxic T cell immune responses against the RBD, protective B cell Ab responses are also dependent on the robust CD4+ T cell activation and cytokine production. Hence, antiviral Ab production in both immunised and infected individuals is correlated to the virus-specific T cell responses. SARS-CoV-2–specific T cell responses were found to be positively correlated with the B cell IgG and nAb responses to the RBD [68]. By utilising RBD as a vaccine candidate, both the humoral and cell-mediated immune responses can be anticipated.

However, employing RBD in a vaccine is compromised due to its limited immunogenicity owing to its small molecular size [34]. Strategies to heighten vaccine-induced immune responses by increasing the antigen size are under investigation. For instance, the Anhui Zhifei Longcom Biopharmaceutical Co., Ltd. (Hefei, China) developed a 60 kDa RBD homodimer by cloning 2 copies of RBD-encoding gene fragments into the tandem RBD monomer, which has a molecular mass of approximately 29 kDa [67]. Meanwhile, Wang et al. successfully designed a chimeric MERS-CoV vaccine by fusing the canine provirus VP2 structural protein gene with the MERS-CoV RBD [69]. This chimeric RBD vaccine induced both humoral and cell-mediated immune responses and demonstrated a high safety profile. With appropriate structural-based modifications and improvements, an RBD-based vaccine may confer higher protective capacity with a lower risk of ADE compared to existing full-length S protein vaccines [70].

In addition to the RBD, the NTD was also investigated for its potential to be the target of a SARS-CoV-2 vaccine. The recombinant MERS-CoV virus NTD vaccine successfully primed mouse B cells to secrete a significant titre of antigen-specific Ab and nAb, but of lower IgG titre than when immunised with the RBD vaccine [71]. Against SARS-CoV-2, the NTD vaccine induced a weaker immune response and lower titre of nAb than that of RBD [72]. Chi et al. identified a naturally occurring human monoclonal Ab, 4A8, exhibiting high neutralisation potency against the natural and pseudotyped SARS-CoV-2 virus present on the NTD [72]. They determined the location, position and structure of the epitope of 4A8 with cryo-EM. Instead of inhibiting the ACE2–S protein interaction, this 4A8 nAb is speculated to confer protective efficacy by restraining the pre-fusion to a post-fusion conformational change of the S protein upon virus docking to a host cell. This study not only revealed a promising vaccine candidate but also a strategy to suppress the viral interaction with the host receptor that is independent of receptor-binding inhibition.

The NTD is capable of eliciting effective functional T cell immunity, even greater than the RBD. A recombinant NTD vaccine evoked T-helper (Th) 1, Th2 and Th17 cell-mediated immunity [73] and also resulted in reduced lung abnormalities compared to recombinant RBD-vaccinated, MERS-CoV-challenged mice [71].

In addition to the RBD and NTD of the S1 subunit, the S2 subunit was also examined as a potential SARS-CoV2 vaccine candidate. Zhao et al. revealed that 6 of 20 T cell epitopes in the S protein are located on the S2 subunit [68]. These epitopes successfully elicited protective T cell immune responses. Since the protective humoral immune response naturally wanes over time, the virus-specific T cell memory response is crucial for long-term protection against viral infections and may be used to evaluate the duration of vaccine-induced protection. S2 was also reported to induce high total IgG-mediated anti-viral protection but only a small proportion of it was neutralising [10,20], hypothesised to be due to the shielding of the S2 subunit by the S1 subunit, resulting in limited S2 subunit exposure to the immune effector cells, especially at the membrane fusion site. Rabbits immunised with the SARS-CoV-2 S2 protein demonstrated lower nAb titres than those administrated with an S1 or RBD vaccine [74]. In addition to lower nAb titres, Wec et al. reported that the nAbs targeting the S2 subunit isolated from convalescent patients showed weaker neutralising potency against the virus than the RBD-targeting nAbs [75]. Additionally, Guo et al. reported incidences of Th2-biased immune response (suspected to potentially mediate ADE) mediated by the recombinant S2 fragment, instead of the anticipated Th1 response [65]. All of these studies suggest that the S2 subunit alone may not be an effective vaccine candidate to elicit favourable B cell responses against the SARS-CoV-2 virus but could be a part of a cocktail vaccine as suggested by Chi et al. [72].

## 7. Pan-Coronavirus Vaccine

The prolonged COVID-19 pandemic argues that a universal coronavirus vaccine should be pursued. Over the last 15 years, several pathogenic human coronaviruses have posed enormous threats to global health with their high transmissibility and severe lower respiratory tract infection, resulting in high morbidity and mortality rates [58]. In preparation against the emerging and re-emerging coronaviruses and their variants, the development of a broad-spectrum, pan-coronavirus vaccine against conserved epitopes is vital to prevent another epidemic or pandemic from occurring [59]. If a broad-spectrum vaccine against the viral S protein is developed, it is speculated to be effective against all the coronaviruses that gain entry into the host cell via the interaction with the ACE2 receptor. Since the S protein is known to confer to infectivity and tropism of the coronaviruses [10], designing a universal coronavirus vaccine targeting the epitopes within the S protein is plausible.

The first pan-coronavirus candidates are the S1 subunit and the RBD within S1. On top of their capacity to elicit a strong immune response, both contain highly conserved amino acid sequences and are relatively homologous among various coronavirus strains [28]. It was revealed that the SARS-CoV-1 and SARS-CoV-2 RBDs confer to 74% genetic homogeneity [60], and their S1 subunits share approximately 50 identical amino acid sequences, out of a total of 681 amino acids [61]. The majority of these identical amino acid sequences were found in the RBDs of their respective S protein. Poh et al. computationally predicted two immunogenic B cell epitopes within the SARS-CoV-2 S protein and studied them against the SARS-CoV-1 S protein [76]. One of them (S14P5) was SARS-CoV-2-specific, located in proximity to the RBD. Ab binding to the S14P5 sterically hindered the RBD association with the ACE2 receptor, neutralising the SARS-CoV-2 virus. Another IgG-dominant region identified was S21P2, a pan-coronavirus target located at the fusion peptide. Ab bound to S21P1 was hypothesised to inhibit the fusion of the host cell plasma membrane and viral envelope, therefore preventing viral entry.

The S2 subunit should also be considered for a pan-coronavirus candidate. Even though the S2 subunit was reported to elicit a weaker neutralising immune response compared to the S1 subunit [65], it has a highly conserved sequence among different human coronavirus species [75], offering the superior potential for a pan-coronavirus vaccine. Additional reports concluded that the S2 fusion domains of SARS-CoV-1 and SARS-CoV-2 viruses share 90% identity [60]. The S2 subunit comprises two highly conserved HR1 and HR2, which confer the production of broadly neutralising Ab against a diversity of coronavirus strains [37,62]. These HRs contribute to viral entry by bringing the virus and host cell together and mediating their membrane fusion [58]. Despite the fact that both HRs are reported to be composed of highly conserved epitopes in various coronaviruses [62], HR2 is deemed to be more promising because it has an identical amino acid sequence in both SARS-CoV-1 and SARS-CoV-2 [63]. Considering the conservation of protein sequence and its contribution to viral entry, HR2 is a promising pan-coronavirus vaccine candidate [37,59]. Furthermore, S2 is more stable than S1 to mutations. In the whole SARS-CoV-1 S protein sequence, 11 amino acid mutation sites were identified and analysed: 9 in the S1 subunit and only 2 in the S2 subunit. These data indicate that the highly conserved S2 subunit is capable of inducing immune protection against various coronavirus strains despite the antigenic drift [65].

Candidates for a broad-spectrum, anti-coronavirus vaccine are not solely limited to the S protein and its subunits. Previous research on vaccines against the influenza virus, which is also capable of rapid evolution [64], may provide a model for constructing a broad-spectrum vaccine against coronaviruses. Some pan-influenza vaccine studies suggest that other highly conserved structural and non-structural viral components elicited appropriate anti-viral immune responses [65]. The M proteins and nucleoproteins of influenza were revealed to be more conserved than the proteins exposed on the viral surface, such as haemagglutinin and neuraminidase. Moreover, Patel et al. reported that the T cell immune response elicited by the conserved influenza epitopes may enhance the broad-spectrum efficacy of the pan-influenza vaccine [66]. Although further investigations are required to examine if these discoveries are applicable in designing a pan-coronavirus vaccine, these studies suggest that the non-S proteins of SARS-CoV-2 may be suitable in stimulating robust anti-viral T cell immune responses and are less susceptible to genetic drift.

Nevertheless, we must note some limitations to the broad-spectrum vaccine. First, the duration of the protective immunity elicited by the broad-spectrum vaccine is not properly defined [65]. The incorporation of adjuvants and a clearly established immunisation schedule are required to stimulate effective and long-lasting anti-viral immunity. If the memory of lymphocytes is lost before the emergence of a novel coronavirus, a broad-spectrum vaccine is ineffectual. Additionally, several studies reported that the pan-influenza vaccine confers better protective efficacy against seasonal influenza, rather than pandemic influenza [65,66]. This knowledge implies that a broad-spectrum vaccine may not be suitable against pandemic coronaviruses.

A pan-coronavirus vaccine may not confer full protection against a novel coronavirus strain but still mitigate a pandemic size and pathology [65]. The cross-protective immunity against a diversity of viral strains dampens the potential rate of viral genome mutation. Most importantly, a pan-virus vaccine may limit the transmission of the virus over a longer period of time, in comparison to strain-specific vaccines. Given the high reproduction number of the SARS-CoV-2 infection and the continuous emergence of new virus variants, the long-term control of coronaviral spread with a pan-coronavirus vaccine is essential [4].

## 8. Emerging Variants and Vaccine Adaptation

**Figure 3:**
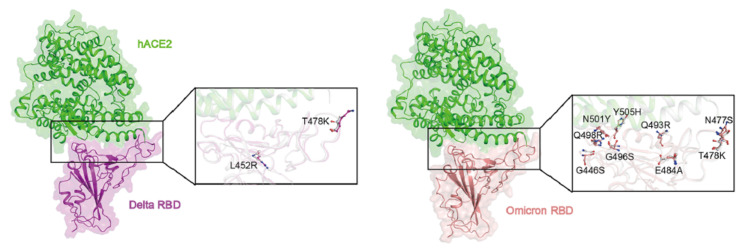
Mutations in the receptor binding domain of the Delta and Omicron variants. The Delta variant acquired key mutations such as L452R and T478K. The Omicron variant obtained important mutations such as Q493R, G496S and Q498R, which contributed to the enhanced infectivity and immune-evading capacity of the virus. Reprinted from Han et al. [86]. The permission to reprint was granted by Cell Press.

The first major SARS-CoV-2 variant was reported in early March 2020, with an amino acid mutation D614G in S protein [77]. Before March 2020, this variant was detected in 10% of 997 global sequences, but by mid-May, it was present in 78% of 12,194 sequences, implying a higher transmission rate than the original strain [78]. The Alpha variant B.1.1.7 was identified in August 2020, in which 8 of 17 mutations were identified in the S protein [79]. The important mutations to note are the N501Y, P681H and 69-HV-70 deletion, which increased the RBD interaction affinity for ACE [80], increased infectivity and transmissibility and facilitated immune escape in immunocompromised patients [79]. The Delta variant B.1.617 emerged at the end of 2020 and contained a pair of mutations (L452R and E484Q) in the RBD associated with improved infectivity and greater affinity to the ACE2 receptor (Figure 3) [81]. The Delta variant is known to be twice as contagious as the previous SARS-CoV-2 variants. The most recently confirmed Omicron variant (B.1.1.529) was shown to have a high capacity for transmission and breakthrough infection compared to any other variants [82] due to crucial D614G, N501Y and K417N mutations in the S protein [83,84,85].

The vaccines’ protective efficacy is waning off with the emergence of every new variant. Injection of two doses of Novavax’s NVX-CoV2372 vaccine expressing a full-length S protein reported an efficacy estimate of 96.4% against the original strain [84] but only demonstrated approximately 60% protection against the B.1.351 variant [85]. On the other hand, the Pfizer-BioNTech’s two-dose regimen of complete S protein vaccine initially achieved 95% protection against the original strain [54], however, it only offered 87% and 72.1% protective efficacy against the UK B.1.1.7 and South African B.1.351 variants, respectively [87]. Collie et al. further confirmed that the efficacy of the two doses of BNT162b2 mRNA vaccine dropped during the proxy Omicron period [88]. The two-dose Pfizer-BioNTech vaccine was 93% efficient when the Delta variant was dominant, but the same vaccine reported only 70% efficiency against the Omicron variant. Callaway et al. estimated that one to two single nucleotide mutations accumulate in the SARS-CoV-2 variant per month [89].

In conjunction with the worrisome immune evasion capacity of the Omicron, the Centres for Disease Control and Prevention (CDC) in the United States strongly emphasised the importance of vaccination and booster shots 6 months after their initial Pfizer or Moderna series or 2 months after their initial Janssen vaccine so as to maintain the vaccine efficacy against the Omicron variant [90]. The results of an observational study conducted in Israel on the effectiveness of a third dose of the Pfizer vaccine support the CDC’s recommendations. This observational study reported that the third dose vaccine prevented COVID-19-related admission to the hospitals 93% more effectively, prevented severe disease by 92% and prevented COVID-19-related death by 81% compared to the 2 doses of the vaccine administered at least 5 months ago [91]. Additionally, the UK Health Security Agency disclosed that the vaccine efficacy against hospital admission for the Omicron variant four weeks after the first dose of AstraZeneca, Pfizer or Moderna vaccine was only 52%, but the vaccine efficacy increased to 72% upon receiving the second dose [92]. However, its protective efficiency started to wane off 25 weeks after the second injection. Nevertheless, the vaccine efficacy surged to 88% 2 weeks after the booster shot (third dose). These data suggest that the addition of a booster shot into the COVID-19 immunisation schedule is crucial in maintaining the protective efficacy of the currently available SARS-CoV-2 vaccines. Additional boosters and/or adaptations to the vaccines with the aid of structural vaccinology tools are necessary to mitigate the waning of the vaccine efficacy with the emergence of the new variants.

The structural and reverse vaccinology as well as bioinformatics are powerful tools in monitoring the critical mutations acquired by the SARS-CoV-2 variants. The data obtained by employing these tools can be translated into re-designing or updating the currently available vaccines in order to maintain high protective efficacy against the emerging variants. Ford et al. predicted the structural changes in the RBD of the new Omicron mutant using the AlphaFold2 algorithm and concluded that despite the reduction in Ab interaction to its epitopes, the virus is incapable of completely evading the current infection or vaccine-induced immunity [93]. However, the atomic and structural configuration revealed by Ni et al. arrived at a different conclusion: They identified specific mutations on epitopes involved in Ab recognition, implying that the Omicron variant *is* capable of evading infection or vaccine-induced humoral immunity [94]. They confirmed that the structures of the S protein, RBD and NTD domains of the Omicron mutant were largely similar to the original SARS-CoV-2 strain with the aid of high-resolution cryo-EM [94]. The Omicron variant emerged as a variant of concern due to its high infectivity and its ability to evade infection and/or vaccine-induced immunity [95]. The capacity of this variant to evade immunity while retaining its ability to bind to the ACE2 receptor was revealed by Mannar et al., who structurally analysed the formation of new salt bridges and hydrogen bonds between the variant’s RBD and the host ACE2 receptor [95]. They demonstrated that the acquisition of Q493R, G496S and Q498R mutations conferred to the Omicron’s ability to bind to the host receptor and suggested that mutations found in the RBD, the primary target for nAbs, may be the cause of the waning of SARS-CoV-2-specific immunity (Figure 3). These studies highlight that in silico prediction and antigen reverse engineering must be coupled with actual structural evidence of the virus and its proteins to derive a complete understanding of the functional and structural alterations of the antigens. Comprehending the consequences of the mutations identified via computational analysis using structural vaccinology tools is essential in guiding the development of effective therapeutics [95]. Thus, structural vaccinology tools are key to the rapid development and update of the current anti-SARS-CoV-2 vaccines to maintain and/or improve their efficacy against upcoming variants.

## 9. Discussion

Structural vaccinology tools are vital for the development of the current COVID-19 vaccine and other modern vaccines. Structural knowledge of the whole virus, antigen and antigen–Ab complex is mandatory for designing a vaccine that is capable of eliciting favourable antiviral immune responses and preventing the ADE of the disease by minimising the production of non-nAb. An ideal vaccine should induce high titres of nAb, elicit robust Th1-biased immune response, stimulate and maintain long-lasting immunological memory and provide cross-protection among various coronavirus strains and variants. Moreover, structural knowledge is essential to stabilise the malleable antigens to their most favourable conformation with the aim of enhancing their antigenicity and/or masking the non-neutralising epitopes [96]. This strategy is to maximise neutralising epitope presentation and minimise the risk of ADE. Structural vaccinology can be utilised to improve vaccine thermostability, potentially solving the cold-chain problems faced in remote and poorer regions and countries [97]. In order to take full advantage of structural vaccinology tools, it is crucial to acquire new structural and functional insights into both viruses and our immune response against pathogens.

The COVID-19 vaccine-induced immunity is plummeting with the evolution of SARS-CoV-2 variants [92]. Meanwhile, some variants evolve to be highly infectious and transmissible. The reason behind the virus immune evasion while retaining or improving their capacity to bind to the human ACE2 receptor is the acquisition of mutations on its S proteins, especially on the RBD [96]. In the aim to update or develop vaccines against the SARS-CoV-2 variants, structural and reverse vaccinology tools are vital in examining the genetic makeup and the molecular structure of the new variants and their proteins for the proper selection of viral antigens, platform for antigen production and adjuvants to formulate a highly efficacious vaccine. Given the rapid evolution of SARS-CoV-2 variants, the nucleotide vaccine platform may be the most flexible vaccine development platform to adapt the acquired mutations of the new variants to the currently available vaccine. The vaccines should only be redesigned after a thorough structural investigation of the mutated S proteins and the epitope–nAb complex.

## 10. Conclusions

We propose that the booster vaccines should adapt both the wild-type and the mutant S protein or the protein-encoding RNA sequences whenever the coronavirus mutants arise. We should take full advantage of genomic databases, structural biology tools, virus structure prediction systems and predictors of the antigenic determinants in the construction of vaccines against the emerging variants [98]. All these tools in combination will keep us vigilant against the ever-evolving SARS-CoV-2 virus and serve as a powerful asset to predict conformational and linear B and T cell epitopes.

## Figures and Tables

**Table 1:** Summary of the vaccine design of the WHO-approved COVID-19 modern vaccines.

WHO-Approved Vaccines (All against the Spike Protein)		Modern Vaccine Platform	Vaccine Design
Pharmaceutical Company	Research Name		
Pfizer-BioNTech	BNT162b2	mRNA	Full-length S protein stabilised with two proline substitution (K986P and V987P) encapsulated in lipid nanoparticle
Moderna	mRNA-1273	mRNA	Full-length S protein stabilised with two proline substitution (K986P and V987P) encapsulated in lipid nanoparticle
Novavax	NVX-CoV2373	Protein subunit	Full-length S protein stabilised with two proline substitution (K986P and V987P) and 682-QQAQ-685 mutation at S1/S2 cleavage site formulated with Matrix-M adjuvant
Janssen	JNJ-78436735 (Ad26.COV2.S)	Non-replicating viral vector (Adenovirus)	Full-length S protein stabilised with two proline substitution (K986P and V987P) contained in replication-deficient adenovirus vector Ad26
Oxford-AstraZeneca	AZD1222 (ChAdOx1)	Non-replicating viral vector (Adenovirus)	Full-length, wild-type S protein with the human tissue plasminogen activator gene leader added at the N-terminus contained in replication-deficient chimpanzee adenovirus vector ChAdOx1

## Data Availability

No new data were created or analyzed in this study. Data sharing is not applicable to this article.
